# Vascular Mechanisms in the Etiology of Hemifacial Microsomia: A Systematic Review of Epidemiological, Clinical, and Genetic Evidence

**DOI:** 10.1002/bdr2.70081

**Published:** 2026-06-20

**Authors:** Karl Jacobs, Aarya Baramakeh, Hedwig van der Meer, Hans Korfage, Bernadette de Bakker, Geerling Langenbach, Roelof Jan Oostra, Frank Lobbezoo

**Affiliations:** ^1^ Department of Oral Pain and Dysfunction, Section Orofacial Anatomy, Academic Centre for Dentistry Amsterdam (ACTA) University of Amsterdam and Vrije Universiteit Amsterdam Amsterdam the Netherlands; ^2^ Department of Medical Biology, Section Clinical Anatomy & Embryology Amsterdam UMC, University of Amsterdam Amsterdam the Netherlands; ^3^ Amsterdam Reproduction and Development Research Institute Amsterdam the Netherlands; ^4^ SOMT University of Physiotherapy Amersfoort the Netherlands; ^5^ Department of Obstetrics and Gynecology, Amsterdam Reproduction & Development Research Institute Amsterdam UMC, University of Amsterdam Amsterdam the Netherlands; ^6^ Department of Pediatric Surgery Erasmus MC – Sophia Children's Hospital, University Medical Center Rotterdam Rotterdam the Netherlands; ^7^ Department of Orofacial Pain and Jaw Function, Faculty of Odontology Malmö University Malmö Sweden

**Keywords:** craniofacial microsomia, hemifacial microsomia, neural crest cells, pharyngeal arch development, vascular disruption, vasculogenesis

## Abstract

**Background:**

Hemifacial microsomia (HFM) is a congenital craniofacial malformation characterized by unilateral hypoplasia of structures derived from the first and second pharyngeal arches. Although the phenotype is well described, the underlying etiology remains incompletely understood. Disturbances in embryonic vascular development have long been proposed as a potential mechanism contributing to the characteristic unilateral craniofacial phenotype.

**Methods:**

A systematic literature review was conducted following PRISMA guidelines. Searches were performed in PubMed, Embase, Web of Science, and CINAHL to identify studies investigating vascular mechanisms in hemifacial microsomia and related craniofacial microsomia phenotypes. Evidence from epidemiological, clinical, radiological, and genetic studies was synthesized narratively because of methodological heterogeneity.

**Results:**

Of 450 screened records, eight studies met the inclusion criteria. Epidemiological studies identified associations between HFM and maternal factors potentially affecting embryonic perfusion, including vasoactive medication exposure, maternal diabetes, multiple gestations, and second‐trimester vaginal bleeding. Population‐based data further demonstrated an increased prevalence of single umbilical artery and growth restriction among affected infants. Clinical and radiological observations described regional vascular abnormalities, including carotid artery attenuation. One genetic association study suggested interactions between pathways involved in vascular development and neural crest cell biology.

**Conclusions:**

The available evidence is consistent with a possible contribution of disturbances in vascular development to the pathogenesis of hemifacial microsomia, particularly in unilateral presentations. However, current evidence remains indirect and heterogeneous. Vascular mechanisms may interact with genetic susceptibility and neural crest cell biology within a broader multifactorial developmental framework. Further studies integrating genomic analyses, developmental models, and detailed vascular imaging are needed to clarify the role of vascular mechanisms in craniofacial microsomia.

## 1 Introduction

Hemifacial microsomia (HFM) is a congenital craniofacial malformation characterized by unilateral hypoplasia of structures derived from the first and second pharyngeal arches, including the mandible, maxilla, zygoma, temporal bone, auricular components, and associated soft tissues (Cline et al. 2014; Cousley and Calvert 1997; Rice 2005). Facial asymmetry with skeletal and soft‐tissue hypoplasia is the most consistent clinical feature, although phenotypic expression is highly variable (Cline et al. 2014; Cousley and Calvert 1997). Disruption of early pharyngeal arch development during the first weeks of gestation is considered central to its pathogenesis (Elsten et al. 2020; Glaeser et al. 2020).

Terminology surrounding this condition has evolved. Hemifacial microsomia, craniofacial microsomia (CFM), and first and second pharyngeal arch syndrome are often used interchangeably (Elsten et al. 2020; Liu and Teng 2023). The broader oculo‐auriculo‐vertebral spectrum (OAVS) reflects the wide phenotypic variability, including ocular and vertebral anomalies, while Goldenhar syndrome is often regarded as a more severe manifestation within this continuum rather than a distinct disorder (Logan et al. 2013; Meenan et al. 2014; Tingaud‐Sequeira et al. 2022).

HFM is among the most common craniofacial birth defects after cleft lip and palate (Rice 2005; Tingaud‐Sequeira et al. 2022). Incidence estimates range from approximately 1 in 3500 to 1 in 5600 live births (Cousley and Calvert 1997; Cousley and Wilson 1992; Poswillo 1974; Rice 2005), whereas broader CFM prevalence estimates vary substantially depending on diagnostic criteria (Elsten et al. 2020; Liu and Teng 2023; Thomas et al. 2023; da Rosa et al. 2024). Expanding molecular insights further support the concept of a phenotypic spectrum rather than a single discrete disorder (Luquetti et al. 2020; Niu et al. 2024; Timberlake et al. 2021).

Despite the well‐characterized phenotype, the etiology of HFM remains incompletely understood and appears multifactorial (Glaeser et al. 2020; Thomas et al. 2023; Tingaud‐Sequeira et al. 2022). Most cases occur sporadically, with a recurrence risk of approximately 2%–3% in first‐degree relatives (Cousley and Calvert 1997; Cousley and Wilson 1992). Genetic studies have identified chromosomal abnormalities and copy number variants in a subset of patients, including rearrangements involving the 22q11.2 locus, which contains genes essential for neural crest cell migration and pharyngeal arch development (Glaeser et al. 2020). In addition, single‐gene variants affecting early craniofacial morphogenesis, such as MYT1 and the spliceosomal protein SF3B2, have been reported (Tingaud‐Sequeira et al. 2022). However, these alterations explain only a minority of cases. This observation suggests that additional developmental mechanisms contribute to disease pathogenesis.

Neural crest cell (NCC) disturbance represents an important embryologic framework, given the extensive contribution of NCCs to first and second pharyngeal arch derivatives (Cousley and Wilson 1992; Rice 2005; Sadler and Rasmussen 2010). Impaired migration, proliferation, or differentiation of these cells may contribute to the mandibular and auricular hypoplasia characteristic of HFM.

Notably, the predominantly unilateral presentation of HFM raises an important mechanistic question. Systemic genetic alterations or generalized disturbances in neural crest cell biology would be expected to affect craniofacial development bilaterally. In contrast, the lateralized phenotype typical of HFM suggests that regional developmental influences may play a decisive role in shaping phenotypic expression. Local disturbances in vascular development or perfusion represent a plausible mechanism, as vascular territories are intrinsically spatially restricted and capable of producing asymmetric developmental effects (Cousley and Wilson 1992; Rice 2005; Sadler and Rasmussen 2010).

Vascular development is a fundamental component of embryonic morphogenesis that extends beyond tissue perfusion to regulate organ patterning and growth (Risau 1997; Sivaraj and Adams 2016; Jacobs et al. 2024). During early embryogenesis, vasculogenesis and angiogenesis establish dynamic vascular networks that coordinate oxygen delivery, growth factor distribution, and endothelial–mesenchymal signaling. Key regulators such as vascular endothelial growth factor (VEGF), bone morphogenetic proteins (BMPs), and fibroblast growth factors (FGFs) influence both vascular remodeling and osteogenic differentiation, underscoring a tightly coupled vascular–osteogenic interface (Ramasamy et al. 2014; Grosso et al. 2017). Craniofacial skeletogenesis, whether through intramembranous or endochondral ossification, requires vascular invasion as a critical step in bone formation (Percival and Richtsmeier 2013; Hu and Olsen 2016; Sivaraj and Adams 2016). Disruption of local vascular remodeling during early arch development may therefore alter craniofacial morphogenesis not only through ischemic injury but also through perturbation of instructive vascular–skeletal interactions.

Several maternal and environmental exposures associated with HFM, including diabetes, cigarette smoking, multiple gestation, hypoxia, and exposure to vasoactive or teratogenic agents such as thalidomide, retinoic acid, and mycophenolic acid, are biologically consistent with disturbances in embryonic perfusion or angiogenesis (Elsten et al. 2020; Meenan et al. 2014; Sadler and Rasmussen 2010; Thomas et al. 2023; Tingaud‐Sequeira et al. 2022). Experimental models demonstrate that teratogen‐induced hemorrhage or vascular occlusion affecting the stapedial or carotid systems can reproduce craniofacial defects resembling HFM (Cousley and Wilson 1992; Poswillo 1974; Rice 2005; Sadler and Rasmussen 2010). Clinical observations, including cases arising in surviving twins after intrauterine co‐twin demise, further support a potential ischemic or thromboembolic mechanism (Cousley and Wilson 1992; Setzer et al. 1981).

Despite these observations, the extent to which disturbed vascular development acts as a primary etiologic driver, a secondary modifying factor, or one component within a broader developmental network remains unresolved (Glaeser et al. 2020; Liu and Teng 2023; Sadler and Rasmussen 2010; Thomas et al. 2023; Tingaud‐Sequeira et al. 2022). These mechanisms may overlap and interact during craniofacial development rather than representing entirely separate etiologic processes.

This systematic review therefore aims to critically evaluate the evidence on the role of disturbed vascular development in the etiology of hemifacial microsomia and related craniofacial microsomia phenotypes by synthesizing epidemiological, clinical, and genetic findings, with particular attention to how localized vascular disturbances may contribute to the characteristic unilateral craniofacial phenotype.

## 2 Methods

### 2.1 Reporting and Protocol

This review was conducted and reported in accordance with the Preferred Reporting Items for Systematic Reviews and Meta‐Analyses (PRISMA) statement (Page et al. 2021). The protocol was not prospectively registered.

### 2.2 Information Sources and Search Strategy

A systematic literature search was performed in PubMed, Embase, Web of Science, and CINAHL on September 2, 2025. The search strategy was developed using the Population, Exposure, Outcome, and Study type (PEOS) framework. Controlled vocabulary terms (e.g., MeSH/Emtree) and free‐text keywords related to (P) hemifacial microsomia and related entities (hemifacial microsomia, craniofacial microsomia, Goldenhar syndrome, oculo‐auriculo‐vertebral spectrum) and (E) broader etiologic and mechanistic concepts (e.g., etiology, pathogenesis, mechanisms, developmental abnormalities, and risk factors) were included in the search. During protocol development, explicit vascular‐development and vascular‐disruption terminology was explored; however, these terms were not consistently represented in relevant human studies and therefore vascular relevance was subsequently assessed during title/abstract screening and full‐text eligibility assessment using predefined inclusion criteria focused on vascular developmental mechanisms. The full electronic search strategy for each database is provided in Supporting Information S1 (Supporting Information S1).

### 2.3 Eligibility Criteria

Studies were eligible for inclusion if they met all of the following criteria:

1. Population: human embryos, fetuses, children, or adults diagnosed with hemifacial microsomia (HFM), craniofacial microsomia (CFM), Goldenhar syndrome, or phenotypes within the oculo‐auriculo‐vertebral spectrum (OAVS), with a clearly described case definition;
2. Exposure/etiology of interest: investigation of vascular developmental mechanisms potentially contributing to HFM/CFM/OAVS (e.g., vascular disruption, ischemia, hemorrhage, hypoperfusion, altered angiogenesis/vasculogenesis, or vascular‐related developmental pathways);
3. Outcomes: any mechanistic, histopathologic, imaging‐based, or epidemiologic evidence addressing an association between vascular development/disruption and the occurrence, severity, or phenotypic expression of HFM/CFM/OAVS;
4. Study type: original peer‐reviewed research (e.g., cohort, case–control, cross‐sectional, imaging, embryologic, histologic, or genetic studies). Full text availability was required. Articles were included if published in English or Dutch.

Studies were excluded if they:

1. focused on craniofacial anomalies outside the HFM/CFM/Goldenhar/OAVS spectrum;
2. were animal studies;
3. did not address vascular developmental mechanisms;
4. did not evaluate a defined relationship between vascular factors and HFM/CFM/OAVS; or
5. were secondary literature (narrative reviews, systematic reviews, meta‐analysis), conference abstracts, letters, editorials, or book chapters. Case reports were included only when they provided explicit etiologic evidence relevant to vascular developmental mechanisms; purely descriptive case reports were excluded.

No restrictions on publication year or study design were applied at the search stage.

### 2.4 Study Selection

**FIGURE 1:**
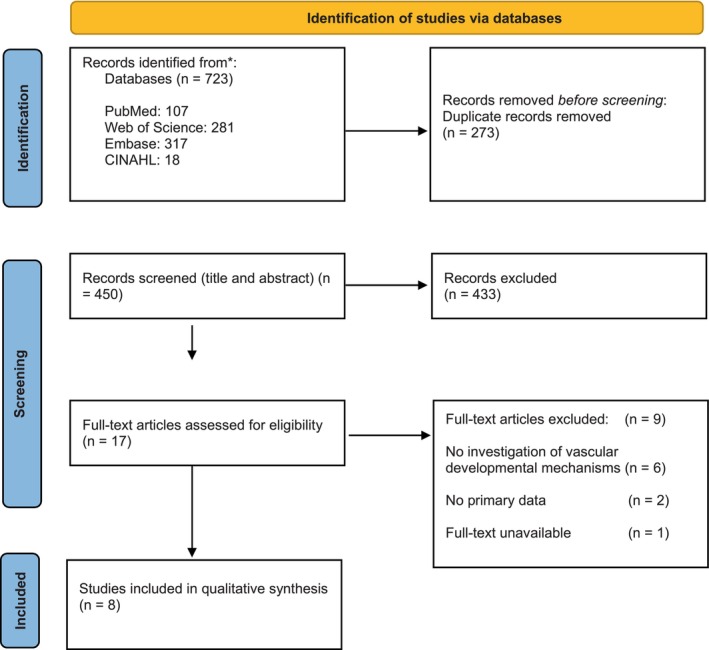
PRISMA flow diagram illustrating the study selection process. Records were identified through systematic searches in PubMed, Embase, Web of Science, and CINAHL. *Source:* Adapted from the PRISMA 2020 flow diagram (Page et al. 2021).

All records retrieved from the database searches were imported into a reference manager for deduplication. Duplicates were identified automatically and verified manually. Title and abstract screening, followed by full‐text screening, were conducted independently by two reviewers (KJ & AB) using Rayyan. Disagreements were resolved by discussion and consensus, with involvement of a third reviewer (HvdM) when necessary. Reasons for exclusion at the full‐text stage were recorded and provided in Table S1. One study (Schinzel et al. 1979) lacked a clearly defined methodological design and was included for contextual interpretation but was not subjected to formal risk of bias assessment. Historical and conceptual studies relevant to the vascular disruption hypothesis (e.g., Poswillo 1974) were cited for contextual interpretation in the Introduction and Discussion but were not included in the formal systematic synthesis when they did not meet the predefined eligibility criteria. One potentially relevant article was excluded because the full text could not be obtained through available institutional resources. The study selection process is presented in a PRISMA flow diagram (Figure 1).

### 2.5 Data Collection and Extraction

Data extraction focused on information relevant to vascular etiology in HFM/CFM/OAVS. A standardized extraction form was used to collect predefined variables: study characteristics (year, country, design, sample size, diagnostic terminology), participant characteristics (age, phenotype/laterality, syndromic features when reported), and vascular‐etiology variables including proposed mechanism(s), embryologic timing, implicated vessels (e.g., stapedial/carotid systems when specified), hemodynamic/perfusion findings, and the nature of supporting evidence (epidemiologic association, imaging/angiography, histopathology, embryologic inference, or genetic findings linked to vascular pathways). Data extraction and verification were performed by two reviewers (A.B. and K.J.).

### 2.6 Study Risk of Bias Assessment

Methodological quality of included studies was assessed using the Joanna Briggs Institute (JBI) Critical Appraisal Tools. The specific tool for each study design (e.g., case–control, cohort, cross‐sectional, or case report) was applied. All tools can be downloaded from the JBI website (https://jbi.global/critical‐appraisal‐tools), which includes checklists and instructions. Each critical appraisal tool consisted of 8 (cross‐sectional study design) to 11 (cohort study design) items. Each item was scored as “yes” (1 point) or “no” (0 points), where a higher total score indicated a lower risk of bias. Critical appraisal was performed independently by two reviewers (AB and KJ), with discrepancies resolved through discussion, or with the help of a third reviewer (HvdM). Appraisal results were used to support interpretation of the strength and limitations of the evidence and to inform the synthesis, rather than to exclude studies. Importantly, JBI appraisal scores were interpreted as indicators of methodological reporting quality and completeness rather than direct measures of mechanistic or causal evidentiary strength.

### 2.7 Synthesis of Results

Given substantial heterogeneity in study design, phenotypic definitions, exposures, and outcome measures, quantitative meta‐analysis was not undertaken. Instead, a narrative synthesis approach was used. Evidence was grouped thematically into three principal domains (epidemiologic associations, clinical/radiologic observations, and genetic/integrative findings). Within each domain, findings were summarized with attention to consistency, specificity to vascular mechanisms, and methodological limitations identified during critical appraisal.

## 3 Results

A total of 723 studies were identified from four databases. After removing 273 duplicates, 450 records were screened, of which 17 were assessed for full‐text eligibility. Of these, 8 were included and 9 were excluded for the following reasons: lack of relevant vascular mechanistic data (*n* = 6), no primary data (*n* = 2), or full‐text unavailable (*n* = 1).

### 3.1 Study Characteristics

Eight studies met the predefined eligibility criteria and were included in the final analysis (Table 1), comprising epidemiological studies, clinical case reports and series, a population‐based surveillance study, a twin analysis, and a genomic association study. Of the eight included studies, three provided epidemiological evidence, including two case–control studies and one population‐based study; two were clinical case reports or case series, one was a twin analysis, one was a genomic association study, and one described a developmental presentation inconsistent with a purely localized vascular mechanism. Collectively, these studies explored potential associations between vascular disturbances and the development of hemifacial microsomia (HFM) or related craniofacial microsomia (CFM) phenotypes across multiple levels of evidence.

**TABLE 1:** Characteristics of studies included in the systematic review.

References	Study design	Population/Sample size	Evidence domain	Main findings relevant to vascular hypothesis
Schinzel et al. (1979)	Epidemiologic analysis of monozygotic twins	660 like‐sexed twin pairs with structural defects, including 385 presumed monozygotic twin pairs from the British Columbia Registry	Developmental disruption	Suggested HFM may occur as a disruptive defect in surviving twins after co‐twin demise, potentially due to thromboembolic or vascular events.
Robinson et al. (1987)	Case series	3 unrelated children with unilateral craniofacial defects	Clinical anatomical/vascular imaging	Documented absent or diminished carotid arterial pulsation and associated anomalies consistent with vascular disruption.
Werler et al. (2004a)	Case–control study	239 HFM cases and 854 controls	Epidemiological	Identified increased risks associated with multiple gestations, second‐trimester vaginal bleeding, low birth weight, and vasoactive‐related reproductive factors. Co‐occurring anomalies included cardiac defects (21%), oral clefts (15%), vertebral anomalies (13%), and ocular abnormalities (14%).
Werler et al. (2004b)	Case–control study	230 HFM cases and 678 controls	Epidemiological	Reported associations between vasoactive medication exposure, maternal diabetes, multiple gestations, and increased HFM risk. Combined exposure to vasoactive medications and cigarette smoking was associated with a 4.2‐fold increased risk.
Magge et al. (2015)	Case report	1 patient with expanded‐spectrum hemifacial microsomia and multisystem anomalies	Developmental/alternative mechanism	Supporting a broader developmental mechanism beyond a purely localized vascular insult.
Zhang et al. (2016)	Genome‐wide association study	939 CFM cases and 2012 controls in the discovery cohort; replication in an additional 443 cases and 1669 controls	Genetic/developmental	Identified susceptibility loci enriched for pathways involved in both neural crest cell biology and vasculogenesis. EPAS1 was highlighted because of its expression in pharyngeal arch tissues and vascular endothelial cells.
Thomas et al. (2023)	Population‐based retrospective study	63 cases of craniofacial microsomia	Epidemiological	Found increased prevalence of single umbilical artery, congenital heart defects, fetal growth restriction, and multiple gestations, suggesting involvement of vascular developmental disturbances.
Singh et al. (2024)	Case report	1 patient with Goldenhar syndrome	Clinical/radiological	Radiological evidence of severe carotid artery attenuation and cerebral hemi‐atrophy consistent with vascular insufficiency.

### 3.2 Risk of Bias in Studies

Overall, the methodological quality of the included studies was variable. Epidemiological studies generally demonstrated moderate methodological quality, whereas case reports and case series were inherently limited by small sample size and lack of control groups. Although several case reports fulfilled multiple JBI reporting criteria, these study designs were nevertheless interpreted as inherently low‐level evidence for causal inference and mechanistic interpretation. No studies were excluded based on methodological quality, and the risk of bias assessment was used to inform the synthesis and support cautious interpretation of the available evidence. The results of the critical appraisal are provided in Table S2.

### 3.3 Results of Individual Studies

Table 1 provides an overview of the included studies, including study design, population, and key findings, categorized across the main evidentiary domains relevant to the vascular hypothesis.

### 3.4 Results of Syntheses

The available evidence addressed three principal domains: (1) epidemiological risk factors associated with vascular compromise during pregnancy, (2) clinical and radiological observations suggestive of localized vascular insufficiency, and (3) genetic findings indicating potential interactions between vascular development and neural crest cell biology.

#### 3.4.1 Epidemiological Evidence Suggestive of Vascular Involvement

Several epidemiological studies have examined maternal exposures and obstetric conditions associated with hemifacial microsomia and identified factors consistent with impaired embryonic perfusion (Table 1). In two case–control studies, Werler and colleagues reported increased risks associated with maternal exposure to vasoactive medications during early pregnancy (odds ratio [OR] 1.9), with the risk rising to OR 4.2 when combined with maternal cigarette smoking (Werler et al. 2004a, 2004b). These findings suggest a possible interaction between vasoactive and hypoxic mechanisms during early craniofacial development.

Additional obstetric factors associated with altered uteroplacental circulation were also identified. Multiple gestations were associated with a markedly increased risk of HFM (OR 9.4), while second‐trimester vaginal bleeding showed an even higher risk estimate (OR 10.5) (Werler et al. 2004a). Maternal diabetes was similarly associated with elevated risk (OR 6.0), suggesting that metabolic or vascular disturbances during early embryogenesis may contribute to the development of HFM (Werler et al. 2004b).

Population‐based surveillance data provide additional support for this association (Table 1). In a retrospective analysis of 63 cases of craniofacial microsomia within the Alberta Congenital Anomalies Surveillance System, Thomas et al. (2023) reported a substantially increased prevalence of single umbilical artery (12.7%) compared with the reported baseline prevalence of approximately 0.5% in the general population. Multiple gestations were also overrepresented among affected pregnancies (12.7% vs. 3.3% in the general population). In addition, 25.4% of affected infants were small for gestational age compared with 9.7% in the general population (*p* = 0.0054). Congenital heart defects were observed in 33.3% of cases, further suggesting that broader disturbances in embryonic vascular development may be associated with craniofacial microsomia.

#### 3.4.2 Clinical and Anatomical Observations Consistent With Vascular Disruption

Clinical observations provide additional evidence consistent with localized vascular insufficiency in some patients with craniofacial microsomia (Table 1). Robinson et al. (1987) described several cases of unilateral craniofacial anomalies associated with diminished or absent carotid arterial pulsation on the affected side. Diagnostic investigations, including carotid phonoangiography and postmortem arteriography, demonstrated abnormalities in the internal, common, or external carotid arteries. In some patients, these vascular findings occurred alongside additional anomalies classically attributed to vascular disruption, such as descending colonic stenosis.

More recent radiological observations provide similar indications of localized vascular compromise. Singh et al. (2024) reported a patient with Goldenhar syndrome presenting severe attenuation of the ipsilateral carotid artery and associated cerebral hemiatrophy. These findings were interpreted as evidence of vascular insufficiency affecting craniofacial and cerebral development on the affected side.

Observations from twin studies further support the possibility of a disruptive vascular mechanism. Schinzel et al. (1979) identified hemifacial microsomia among structural anomalies occurring in surviving twins following intrauterine death of the co‐twin. In such cases, intravascular coagulation or thromboembolic events have been proposed as potential mechanisms producing localized ischemic injury during embryonic development. Additionally, the low concordance rates observed among monozygotic twins affected by the facio‐auriculo‐vertebral spectrum have been interpreted as supporting a non‐genetic disruptive process.

#### 3.4.3 Evidence Suggesting Alternative or Interacting Mechanisms

Although several studies are consistent with a vascular contribution to craniofacial microsomia, other findings suggest that additional developmental mechanisms may also be involved (Table 1). A case report describing an expanded‐spectrum presentation of hemifacial microsomia reported widespread bilateral anomalies involving craniofacial, central nervous system, renal, cardiac, and limb structures (Magge et al. 2015). The extent of these abnormalities exceeded what would be expected from a localized vascular insult, suggesting the possibility of earlier developmental disturbances affecting blastogenesis or neural crest cell development.

Genetic studies provide further evidence that multiple developmental pathways may contribute to craniofacial microsomia. A genome‐wide association study identified several susceptibility loci enriched for genes involved in both neural crest cell biology and vascular development (Zhang et al. 2016). Among the identified susceptibility loci, one locus at 2p21 implicated EPAS1, which was highlighted because of its expression in pharyngeal arch tissues and vascular endothelial cells. Although these findings are biologically compatible with vascular developmental mechanisms, direct mechanistic evidence for vascular causality remains limited. These findings suggest that neural crest cell dysfunction and vascular abnormalities may interact during craniofacial development, rather than representing mutually exclusive mechanisms.

Taken together, findings across the three principal domains, epidemiological associations, clinical and radiological observations, and genetic or interacting developmental mechanisms suggest that disturbances in vascular development may contribute to the etiology of hemifacial microsomia in a subset of cases. However, the available evidence also indicates that vascular mechanisms likely interact with broader developmental pathways, including genetic susceptibility and neural crest cell biology.

## 4 Discussion

The findings of this systematic review suggest that disturbances in vascular development may contribute to the pathogenesis of hemifacial microsomia (HFM), although the available evidence remains indirect and methodologically heterogeneous. Several epidemiological studies identified maternal exposures and obstetric conditions associated with impaired uteroplacental or embryonic perfusion, including vasoactive medication use, maternal diabetes, multiple gestations, and second‐trimester vaginal bleeding (Werler et al. 2004a, 2004b). Nicotine‐induced vasoconstriction may further contribute to impaired embryonic perfusion in addition to hypoxia‐related effects. Population‐based data further demonstrated an increased prevalence of single umbilical artery, growth restriction, and congenital heart defects among affected infants (Thomas et al. 2023), supporting the possibility of broader vascular developmental disturbances. Together, these observations suggest that compromised vascular supply during early embryogenesis may represent one pathway contributing to the development of HFM. Importantly, vascular‐associated exposures and developmental findings were not universally present among affected individuals, supporting a multifactorial developmental model in which vascular mechanisms likely interact with additional genetic and developmental influences.

The vascular disruption hypothesis, originally proposed by Poswillo (1974), provides a biologically plausible developmental framework for these observations. According to this model, transient hemorrhagic or ischemic events affecting the developing stapedial artery during a critical window of craniofacial morphogenesis may damage adjacent tissues derived from the first and second pharyngeal arches. Because the stapedial artery represents the principal arterial supply to these structures between approximately gestational days 33 and 45, localized vascular compromise during this period could theoretically result in the characteristic unilateral craniofacial hypoplasia observed in HFM (Robinson et al. 1987). However, direct causal evidence in humans remains limited. Consequently, the vascular hypothesis should be interpreted as a plausible developmental mechanism rather than a definitive causal explanation.

Experimental animal studies, including the work of Poswillo (1974), have historically provided important support for the vascular disruption hypothesis. These models form the historical basis of this concept, demonstrating that vascular perturbations during early development can induce craniofacial anomalies resembling hemifacial microsomia. However, animal studies were not included in the present review, as the analysis was restricted to human data.

Clinical and radiological observations provide additional, though largely anecdotal, support for regional vascular insufficiency. Case reports describing diminished carotid arterial flow or severe carotid attenuation on the affected side demonstrate anatomical correlations between vascular abnormalities and craniofacial asymmetry (Robinson et al. 1987; Singh et al. 2024). Similarly, observations from monozygotic twin studies suggest that disruptive events such as thromboembolism following intrauterine co‐twin demise may produce localized ischemic injury during early development (Schinzel et al. 1979). Although these findings are consistent with a vascular mechanism, their rarity and limited sample sizes prevent definitive conclusions. In addition to vascular explanations, several alternative developmental mechanisms have been proposed for HFM, including genetic susceptibility and disturbances in neural crest cell development.

Importantly, the predominantly unilateral presentation of HFM presents a challenge for etiological models based solely on systemic genetic defects, as genetic variants affecting craniofacial development might generally be expected to produce more bilateral effects. Nevertheless, asymmetric phenotypic expression can occur in genetically mediated craniofacial disorders through mechanisms such as variable expressivity, developmental stochasticity, modifier effects, mosaicism, or gene–environment interactions. Therefore, the lateralized presentation of HFM may be compatible with local vascular or perfusion‐related developmental disturbances, while not excluding a contribution from underlying genetic susceptibility. Notably, detailed clinical and morphometric observations often reveal subtle involvement of the contralateral side, suggesting that the condition may represent an asymmetric bilateral disturbance rather than a strictly unilateral defect. Similar patterns of lateral predominance have been described in other craniofacial malformations, such as cleft lip and palate, indicating that intrinsic left–right differences during craniofacial development may influence phenotypic expression. Within this context, local disturbances in vascular supply may provide one plausible explanation for the observed asymmetry, while not excluding contributions from genetic susceptibility, developmental stochasticity, modifier effects, or gene–environment interactions, potentially amplifying subtle intrinsic differences between the left and right sides of the developing craniofacial complex during early pharyngeal arch development. Nevertheless, vascular disruption alone cannot fully explain the phenotypic diversity observed within the craniofacial microsomia spectrum. While localized vascular disturbances provide a plausible explanation for many typical unilateral presentations of HFM, expanded‐spectrum cases involving bilateral or multisystem anomalies, including cardiac, renal, skeletal, and central nervous system defects, likely reflect earlier or more global developmental disturbances, potentially involving blastogenesis or widespread neural crest cell dysfunction (Magge et al. 2015; Sadler and Rasmussen 2010). The included GWAS study by Zhang et al. (2016) identified susceptibility loci potentially linking vascular development and pharyngeal arch biology. Additional genetic studies from the broader craniofacial developmental literature further support this etiological heterogeneity. Variants in genes such as SF3B2, FOXI3, MYT1, and OTX2 demonstrate that molecular pathways governing pharyngeal arch development may contribute to disease susceptibility (Luquetti et al. 2020; Mao et al. 2023; Timberlake et al. 2021; Zielinski et al. 2014). Notably, several of these pathways intersect with vascular signaling mechanisms, suggesting that genetic susceptibility may influence craniofacial development partly through effects on angiogenesis or vascular stability.

Neural crest cell biology provides an additional mechanistic framework that may interact with vascular development. Cranial neural crest cells (CNCCs) contribute extensively to the skeletal and connective tissues derived from the first and second pharyngeal arches. Their migration, proliferation, and differentiation are regulated by multiple signaling pathways, including BMP, Wnt, FGF, and retinoic acid (Chen et al. 2018; Paul et al. 2020). Disruption of these pathways can lead to craniofacial malformations resembling the phenotypic features of HFM. Importantly, neural crest cells are highly sensitive to their microenvironment, including local oxygen tension and endothelial‐derived signals. Consequently, vascular disturbances and neural crest cell dysfunction should not necessarily be considered competing hypotheses but rather interacting processes within a broader developmental network governing craniofacial morphogenesis.

Interpretation of the current evidence requires consideration of several methodological limitations. The included studies were heterogeneous in design and evidence level, ranging from large case–control studies to single case reports. Epidemiological associations are susceptible to recall bias and exposure misclassification, while case reports provide limited generalizability. In addition, inconsistent terminology across studies, particularly the interchangeable use of HFM, craniofacial microsomia, and Goldenhar syndrome, complicates direct comparison between cohorts. These limitations restrict the ability to draw definitive conclusions regarding causal mechanisms.

Taken together, the available evidence indicates that disturbances in vascular development represent a biologically plausible contributor to the pathogenesis of hemifacial microsomia. Epidemiological associations with maternal vascular risk factors, clinical observations of regional vascular insufficiency, and emerging genetic findings involving pathways related to vasculogenesis collectively support a potential role for impaired embryonic perfusion during early pharyngeal arch development. At the same time, the heterogeneity of the included studies and the absence of direct mechanistic evidence in humans require cautious interpretation within a broader multifactorial framework that also includes neural crest cell biology and genetic susceptibility. Although several case reports and case series fulfilled multiple JBI reporting criteria, these study designs were nevertheless interpreted as inherently low‐level evidence for causal inference and mechanistic interpretation. Rather than representing mutually exclusive explanations, vascular disruption and neural crest cell dysfunction may interact within the complex developmental environment that governs craniofacial morphogenesis. By systematically synthesizing epidemiological, clinical, and genetic evidence, this review provides a developmental framework linking localized vascular disturbances to the characteristic unilateral phenotype of hemifacial microsomia.

## 5 Conclusion

This systematic review suggests that disturbances in vascular development may contribute to the pathogenesis of hemifacial microsomia, particularly as a possible explanation for the characteristic unilateral phenotype. While epidemiological associations and clinical observations provide indirect support, the available evidence remains limited and heterogeneous, and direct confirmation in humans is lacking. These findings support a multifactorial developmental model in which vascular disturbances may interact with genetic susceptibility and neural crest cell biology. Future research integrating genomic data, detailed vascular imaging, and experimental developmental models will be essential to clarify the relative contribution of vascular mechanisms within the broader etiological framework of craniofacial microsomia.

## Funding

The authors have nothing to report.

## Ethics Statement

The authors have nothing to report.

## Conflicts of Interest

The authors declare no conflicts of interest.

## Supporting information


**Supporting Information S1:** Full electronic search strategies for all databases.

**Table S1:** Full‐text articles excluded after eligibility assessment with reasons.

**Table S2:** Risk of bias assessment of included studies (JBI Critical Appraisal Tools).

## Data Availability

The data that support the findings of this study are available within the article and its Supporting Information.
