# Supplementary material for: Vascular Mechanisms in the Etiology of Hemifacial Microsomia: A Systematic Review of Epidemiological, Clinical, and Genetic Evidence
Source: Birth Defects Res. 2026 Jun 20;118(6):e70081. doi: 10.1002/bdr2.70081 (PMC13282715; doi:10.1002/bdr2.70081)
Supplement: Supplementary file 1 — Supporting Information S1: Full electronic search strategies for all databases. [file BDR2-118-e70081-s001.docx]

## Supplementary Material S1. Full electronic search strategies for all databases

### PubMed

**Database:** PubMed
**Date of search:** 2 September 2025

("craniofacial microsomia"[MeSH Terms] OR "goldenhar syndrome"[MeSH Terms] OR "goldenhar disease"[MeSH Terms] OR "hemifacial microsomia"[MeSH Terms])

AND

("etiology"[Title/Abstract] OR "causation"[Title/Abstract] OR "pathogenesis"[Title/Abstract] OR "mechanisms"[Title/Abstract] OR "risk factors"[Title/Abstract] OR "determinants"[Title/Abstract] OR "contributing factors"[Title/Abstract] OR "pathophysiology"[Title/Abstract] OR "disease mechanism"[Title/Abstract] OR "developmental causes"[Title/Abstract])

**Records retrieved:** 107

### Embase

**Database:** Embase
**Date of search:** 2 September 2025

('goldenhar syndrome'/exp OR 'craniofacial microsomia'/exp OR 'hemifacial microsomia'/exp)

AND

(etiology:ab,ti OR causation:ab,ti OR pathogenesis:ab,ti OR 'underlying factors':ab,ti OR mechanisms:ab,ti OR 'risk factors':ab,ti OR determinants:ab,ti OR 'contributing factors':ab,ti OR pathophysiology:ab,ti OR 'disease mechanism':ab,ti OR 'developmental causes':ab,ti)

**Records retrieved: 317**

### Web of Science

**Database:** Web of Science
**Date of search:** 2 September 2025

TS=("goldenhar syndrome" OR "goldenhar disease" OR "hemifacial microsomia" OR "craniofacial microsomia")

AND

TS=("etiology" OR "causation" OR "pathogenesis" OR "mechanisms" OR "risk factors" OR "determinants" OR "contributing factors" OR "pathophysiology" OR "disease mechanism" OR "developmental causes")

**Records retrieved:** 281

### CINAHL

**Database:** CINAHL
**Date of search:** 2 September 2025

(SU "goldenhar syndrome" OR SU "craniofacial microsomia" OR SU "hemifacial microsomia")

AND

(TI etiology OR AB etiology OR TI causation OR AB causation OR TI pathogenesis OR AB pathogenesis OR TI mechanisms OR AB mechanisms OR TI "risk factors" OR AB "risk factors")

**Records retrieved:** 18

Broad etiological and mechanistic terminology was intentionally used to maximize search sensitivity, as studies relevant to vascular developmental mechanisms were frequently discussed indirectly and not consistently indexed using explicit vascular terminology.
