# Supplementary material for: Vascular Mechanisms in the Etiology of Hemifacial Microsomia: A Systematic Review of Epidemiological, Clinical, and Genetic Evidence
Source: Birth Defects Res. 2026 Jun 20;118(6):e70081. doi: 10.1002/bdr2.70081 (PMC13282715; doi:10.1002/bdr2.70081)
Supplement: Supplementary file 2 — Table S1: Full‐text articles excluded after eligibility assessment with reasons. [file BDR2-118-e70081-s002.docx]

## Supplementary Table S1. Full-text articles excluded after eligibility assessment with reasons

Full-text articles excluded after eligibility assessment (n = 9): one article was excluded because the full text was unavailable, two articles were excluded because they did not contain primary data, and six articles were excluded based on predefined eligibility criteria.

| Author(s) & Year | Article Title | Reasons for Exclusion |
| --- | --- | --- |
| Poswillo (1974) | Otomandibular Deformity: Pathogenesis as a Guide to Reconstruction | Primarily reconstructive focus; no primary embryologic vascular pathway analysis |
| Setzer et al. (1981) | Etiologic Heterogeneity in the Oculoauriculovertebral Syndrome | Discusses etiologic heterogeneity; no vascular developmental mechanism evaluated |
| Cousley & Wilson (1992) | Hemifacial Microsomia: Developmental Consequence of Perturbation of the Auriculofacial Cartilage Model? | Focuses on chondrogenesis model; vascular etiology not examined |
| Werler et al.  (2009) | Hemifacial microsomia: From gestation to childhood | Secondary literature; no primary data addressing vascular mechanisms in HFM |
| Sadler & Rasmussen (2010) | Examining the Evidence for Vascular Pathogenesis of Selected Birth Defects | Narrative review; no primary data addressing vascular mechanisms in HFM |
| Paliga et al. (2015) | Cranial Base Deviation in Hemifacial Microsomia by Craniometric Analysis | Anatomical outcome study; does not assess primary vascular etiology |
| Toufaily et al. (2018) | Causes of Congenital Malformations | Broad epidemiologic report; no HFM-specific vascular mechanism analysis |
| Wang et al. (2023) | Discrepancy in Mandibular Medullary Cavity on Different Sides: More Hints Towards Understanding Hemifacial Microsomia | Morphological analysis only; no investigation of embryonic vascular developmental mechanisms |
