# Supplementary material for: Vascular Mechanisms in the Etiology of Hemifacial Microsomia: A Systematic Review of Epidemiological, Clinical, and Genetic Evidence
Source: Birth Defects Res. 2026 Jun 20;118(6):e70081. doi: 10.1002/bdr2.70081 (PMC13282715; doi:10.1002/bdr2.70081)
Supplement: Supplementary file 3 — Table S2: Risk of bias assessment of included studies (JBI Critical Appraisal Tools). [file BDR2-118-e70081-s003.docx]

Supplementary Table S2. Risk of bias assessment of included studies (JBI Critical Appraisal Tools).

| Author (Year) | Study design | JBI checklist | Satisfied (n) | Unmet (n) | Unclear (n) | Not applicable (n) | Score (%) | Quality rating |
| --- | --- | --- | --- | --- | --- | --- | --- | --- |
| Robinson et al. (1987) | Case series | JBI Case Series | 7 | 0 | 2 | 1 | 70% (7/10) | Moderate |
| Werler et al. (2004a) | Case–control | JBI Case-Control | 7 | 3 | 0 | 0 | 70% (7/10) | Moderate |
| Werler et al. (2004b) | Case–control | JBI Case-Control | 7 | 3 | 0 | 0 | 70% (7/10) | Moderate |
| Magge et al. (2015) | Case report | JBI Case Report | 8 | 0 | 0 | 0 | 100% (8/8) | High |
| Zhang et al. (2016) | Genetic association (GWAS) | JBI Analytical cross-sectional | 10 | 0 | 0 | 0 | 100% (10/10) | High |
| Thomas et al. (2023) | Population-based retrospective (prevalence) | JBI Prevalence | 4 | 1 | 2 | 1 | 50% (4/8) | Moderate |
| Singh et al. (2024) | Case report | JBI Case Report | 7 | 0 | 0 | 1 | 87.5% (7/8) | High |

REFERENCES
Paliga, J. T., Y. Tahiri, J. Wink, S. P. Bartlett, and J. A. Taylor. 2015. “Cranial Base Deviation in Hemifacial Microsomia by Craniometric Analysis.” Journal of Craniofacial Surgery 26, no. 1: e61–e64. <https://doi.org/10.1097/SCS.0000000000001182>

Toufaily, M. H., M. N. Westgate, A. E. Lin, and L. B. Holmes. 2018. “Causes of Congenital Malformations.” Birth Defects Research 110, no. 2: 87–91. <https://doi.org/10.1002/bdr2.1105>

Wang, X., H. Xu, B. S. Kim, Y. Zhang, and G. Chai. 2023. “Discrepancy in Mandibular Medullary Cavity on Different Sides: More Hints Towards Understanding Hemifacial Microsomia.” Journal of Craniofacial Surgery 34, no. 2: 575–579. <https://doi.org/10.1097/SCS.0000000000008810>

Werler, M. M., J. R. Starr, Y. K. Cloonan, and M. L. Speltz. 2009. “Hemifacial Microsomia: From Gestation to Childhood.” Journal of Craniofacial Surgery 20, no. Suppl. 1: 664–669. <https://doi.org/10.1097/SCS.0b013e318193d5d5>
